# The reciprocal relationship between gait and handgrip strength across different age groups

**DOI:** 10.3389/fpubh.2025.1557834

**Published:** 2025-04-29

**Authors:** Gayeong Hyeon, Changhun Kim, Sunghoon Shin

**Affiliations:** ^1^Neuromuscular Control Laboratory, Yeungnam University, Gyeongsan, Republic of Korea; ^2^Research Institute of Human Ecology, Yeungnam University, Gyeongsan, Republic of Korea; ^3^Institute of Sports Science, Yeungnam University, Gyeongsan, Republic of Korea

**Keywords:** grip strength, gait, age, gait performance, gait variability

## Abstract

**Introduction:**

This study aimed to investigate the reciprocal relationship between grip strength and gait function across different age groups to better understand age-specific physical performance patterns.

**Methods:**

A total of 328 participants were categorized into young (19–39 years), middle-aged (40–59 years), and older adults (60–89 years). Grip strength, spatiotemporal gait parameters, and gait variability were assessed using validated measurement tools. To investigate the reciprocal relationship, hierarchical moderated regression analyses were conducted to assess the effects of grip strength on spatiotemporal gait parameters, considering age as a moderator, whereas stepwise linear regression analyses were performed to evaluate the predictive effects of spatiotemporal gait parameters on grip strength, controlling for age and additional covariates.

**Results:**

Grip strength significantly influenced gait performance variables—stride length, step length, and walking speed—with the strongest effects observed in older adults. However, grip strength did not significantly impact gait variability, which appeared to be primarily affected by age-related neuromuscular changes. Furthermore, this study highlights that gait performance itself may influence grip strength, particularly in older adults, suggesting a reciprocal relationship between upper and lower limb function. Specifically, the proportion of the double support phase—known to increase with age—was found to be a significant predictor of grip strength, likely reflecting compensatory adaptations for balance maintenance under conditions of declining neuromuscular function.

**Discussion:**

These findings suggest that the mechanisms underlying gait performance differ from those related to gait stability. While grip strength may serve as an accessible biomarker for functional ability in older adults, its relevance in younger and middle-aged adults appears limited. Age-specific interventions are recommended: for older adults, grip-strengthening exercises combined with gait stability training may be beneficial; for younger and middle-aged adults, enhancing neuromuscular coordination and flexibility could be more effective in supporting gait function.

## 1 Introduction

Walking significantly influences an individual’s mobility, functional independence, and overall quality of life (1, 2). Limited walking ability can lead to various negative outcomes, such as social isolation and deteriorating physical health, which are particularly severe in older adults (3). Moreover, walking serves not only as a key metric for evaluating physical function but also as a marker for health conditions, allowing the identification of fall risk factors and the effectiveness of clinical interventions (4, 5). Hence, the objective evaluation of walking provides a preventive and effective strategy to maintain physical, mental, and social well-being.

Walking can be quantified using temporal and spatial parameters, which reflect physical function and health status (6). Temporal parameters, such as walking speed and the double support phase, indicate the overall quality and symmetry of the gait (7, 8). Spatial parameters, including step length and stride length, are essential indicators of gait efficiency and stability (7, 9). For instance, the step length indicates the propulsive force during ground contact, while the stride length is associated with maintaining the dynamic balance and reducing the fall risk. To comprehensively assess walking performance, it is critical to consider both the temporal and spatial gait parameters (10, 11).

While walking performance has traditionally been assessed using mean values, recent studies have increasingly used standard deviation and coefficient of variation (CV) to evaluate gait variability (12). Gait variability is closely associated with neuromuscular control and age-related muscle weakening, providing valuable information for fall risk prediction (13). Previous studies have shown that gait variability tends to increase with age, with a greater stride variability directly associated with heightened fall risk (11). These findings suggest that gait variability is a powerful metric for quantifying age-related changes and predicting falls.

Muscle weakness, particularly lower limb weakness, is one of the primary causes of gait impairment (14, 15). Lower limb strength is critical for walking speed, step length, and mobility tasks (16–18) and has been identified as a major risk factor for falls, especially in older adults (19). Weak lower limb strength negatively affects the gait parameters and reduces the ability to respond to disturbances, thereby compromising stability (19, 20). Moreover, the ability to generate power rapidly declines more significantly with age than with maximal strength, suggesting that power generation may play a more critical role in fall prevention. In contrast, younger adults typically maintain higher levels of overall strength and power, which may reduce the impact of grip strength reduction on walking performance (14, 21).

Grip strength serves as a simple yet effective surrogate marker of lower limb strength, reflecting the overall muscle strength (22, 23). It is closely associated with key gait parameters, such as walking speed and stride length, particularly in older adults, where reduced grip strength may lead to impaired gait stability and propulsion (19, 24). Moreover, grip strength is associated with not only walking performance but also fall risk, making it a key metric for assessing physical function (22).

The impact of grip strength on walking performance can vary depending on the specific gait parameters being assessed, as well as the individual’s age. For example, walking speed, a commonly used metric of gait performance, is more sensitive to exercise interventions involving strength training than maximal walking speed, while the ability to generate force rapidly plays a crucial role in preventing falls under unexpected circumstances (19, 25). These findings suggest that the influence of grip strength on gait performance may be modulated by both gait parameters and age. Studies have shown that lower limb strength and grip strength are interrelated (), indicating that age-related declines in muscle strength and power may simultaneously affect both upper and lower limb functions. Among older adults, reduced muscle strength and power may compromise gait stability and propulsion, leading to increased gait variability.

Conversely, the influence of walking on grip strength should also be considered. Walking reflects neuromuscular control, physical activity levels, and balance maintenance, all of which can influence the preservation or decline of grip strength. Limited walking ability may lead to reduced physical activity and accelerated muscle weakening, resulting in a more rapid decline in grip strength (14, 20). In older adults, the interaction between reduced walking ability and declining grip strength is significant, potentially creating a vicious cycle of functional decline. These findings suggest the need to investigate the relationship between walking ability and grip strength from a bidirectional perspective, with a focus on the moderating role of age.

Aging affects neuromuscular function, mobility, and muscle strength, directly impacting gait performance and stability. Public health organizations, including the CDC and WHO, commonly define older adulthood using a cut-off of 60 years (26). This classification is widely applied in gerontology and physical function research to assess age-related changes in mobility and muscle function. Given the acceleration of sarcopenia, neuromuscular decline, and fall risk around this age, the inclusion of 60 years as a cutoff point in age classification was considered to effectively analyze the age-related differences between gait and grip strength in this study.

Although previous research has demonstrated the relationship among lower limb strength, grip strength, and gait performance, this study provides a novel perspective by exploring the bidirectional association between grip strength and gait performance. Compared with conventional studies that primarily investigate the effect of grip strength on walking ability, this study also examines how gait parameters contribute to grip strength maintenance or decline. Moreover, we analyze how these relationships evolve with age, offering insights into age-specific variations among young, middle-aged, and older adults. Moreover, this study addresses a literature gap by evaluating whether grip strength influences gait variability and how these interactions collectively contribute to fall risk and mobility decline. This study also uniquely incorporated gait variability as a major component.

This study intended to enhance prior research by incorporating a dynamic framework of functional relationships. Instead of treating grip strength and gait characteristics as separate metrics, they exhibit interdependent connection and how these correlations evolved with age. This perspective improved our understanding of age-related functional deterioration and provided a foundation for the development of targeted intervention strategies that addressed upper and lower limb functioning. Thus, our findings offered valuable insights for improving rehabilitation and exercise programs to maintain mobility and prevent functional decline in the older adult population.

This study investigates the effect of grip strength on gait performance and variability, specifically examining how age moderates the relationship between grip strength and walking ability. To elucidate the contribution of grip strength to walking performance and stability, the primary focus is to analyze the interactive effects of age and grip strength on gait parameters, such as stride length and double support phase.

Second, it explores the interactive effects of walking parameters and age on grip strength, determining how gait characteristics contribute to maintaining or accelerating the decline in grip strength. For instance, walking speed and stride length may influence grip strength by enhancing or reducing neuromuscular control and overall physical activity levels.

Through the analysis of the reciprocal relationship between grip strength and walking, this study aimed to provide insights into age-specific functional changes and improve the understanding of how neuromuscular control and activity levels interact with grip strength and gait across different age groups.

## 2 Materials and methods

### 2.1 Participants

This study included 328 participants (108 males and 220 females), which were divided into three groups: young (19–39 years; 46 males and 56 females), middle-aged (40–59 years; 32 males and 80 females), and old-aged groups (60–89 years; 30 males and 84 females) (Table 1). The participants were selected from a cohort of healthy seniors residing in the community via public announcements and were chosen based on their capacity for independent ambulation and their readiness to participate in physical exercise. The mean age of the participants was 50 years (50.12 ± 18.08, minimum = 19, maximum = 85), and only those without musculoskeletal disorders or neurological symptoms in the preceding 6 months were included; patients with any form of Chronic Obstructive Pulmonary Disease and dyspnea and those with neuromuscular disorders who underwent the implantation of prosthetic joints or metallic devices were excluded from the study. All subjects engaged in the research after providing consent and being briefed on its goal, process, and precautions. This study was approved by the Yeungnam University Institutional Review Board (IRB-7002016-A-2023-015). Table 1 shows the physical features of the individuals together with the data on the assessed grip strength, body composition, and gait performance.

**Table 1:** Demographic information of the participants.

Characteristics	Young (*n* = 102)	Middle (*n* = 112)	Older (*n* = 114)	Total (*n* = 328)	*p*-value	*Post hoc*
Genders [female *n* (%)]	56 (54.9%)	80 (71.4%)	84 (73.7%)	220 (67.1%)	0.006 *	✝☨
Age (years)	27.27 ± 5.36	50.86 ± 4.85	69.85 ± 5.77	50.13 ± 18.09	< 0.001 *	✝☨₸
Height (cm)	168.22 ± 9.35	163.72 ± 7.40	158.47 ± 7.13	163.30 ± 8.88	< 0.001 *	✝☨₸
Weight (kg)	68.26 ± 14.03	62.99 ± 11.23	61.02 ± 8.52	63.95 ± 11.74	< 0.001 *	✝☨
BMI (kg/m2)	23.97 ± 3.47	23.39 ± 3.11	24.26 ± 2.57	23.87 ± 3.07	NS	NS
BFP (%)	28.22 ± 7.90	29.13 ± 6.81	32.24 ± 6.05	29.93 ± 7.12	< 0.001 *	☨₸
SMM (kg)	27.28 ± 7.18	24.48 ± 5.67	22.36 ± 4.18	24.61 ± 6.07	< 0.001 *	✝☨₸
Grip strength (kg)	32.81 ± 10.77	27.35 ± 8.96	25.53 ± 6.67	28.41 ± 9.37	< 0.001 *	✝☨
Average gait parameters (gait performance)						
Gait speed (m/s)	1.29 ± 0.16	1.40 ± 0.13	1.29 ± 0.16	1.33 ± 0.16	< 0.001 *	✝₸
Double support phase (%)	22.08 ± 3.76	20.40 ± 3.14	22.72 ± 3.70	21.73 ± 3.67	< 0.001 *	✝₸
Stride time (s)	1.06 ± 0.07	0.99 ± 0.06	1.00 ± 0.06	1.02 ± 0.07	< 0.001 *	✝☨
Stride length (m)	1.35 ± 0.12	1.37 ± 0.09	1.27 ± 0.14	1.33 ± 0.13	< 0.001 *	☨₸
Step length (m)	0.68 ± 0.06	0.69 ± 0.05	0.64 ± 0.07	0.67 ± 0.07	< 0.001 *	☨₸
Swing width (m)	−0.04 ± 0.02	−0.04 ± 0.01	−0.04 ± 0.01	−0.04 ± 0.01	NS	NS
Gait variability						
Double support CV (%)	10.09 ± 4.90	9.32 ± 4.41	7.96 ± 4.40	9.09 ± 4.64	0.003 *	☨
Stride time CV (%)	2.53 ± 0.81	2.26 ± 0.67	2.25 ± 0.64	2.34 ± 0.72	0.006 *	✝☨
Stride length CV (%)	3.41 ± 0.79	3.35 ± 0.85	3.41 ± 0.77	3.39 ± 0.80	NS	NS
Step length CV (%)	4.74 ± 1.18	4.39 ± 1.17	4.61 ± 1.25	4.58 ± 1.20	NS	NS
Swing width CV (%)	−33.25 ± 20.88	−31.47 ± 14.26	−31.61 ± 16.67	−32.07 ± 17.33	NS	NS

Data were expressed as mean ± SD. BMI, body mass index; BFP, body fat percentage; SMM, skeletal muscle mass; CV, coefficient of variation. * indicates significant difference (*p* < 0.05). NS, not significant. The p-values are significant differences between the age groups [Scheffé’s Post hoc - Y vs. M: ✝, Y vs. O: ☨, M vs. O: ₸ (*p* < 0.05)].

### 2.2 Body composition

**Figure 1:**
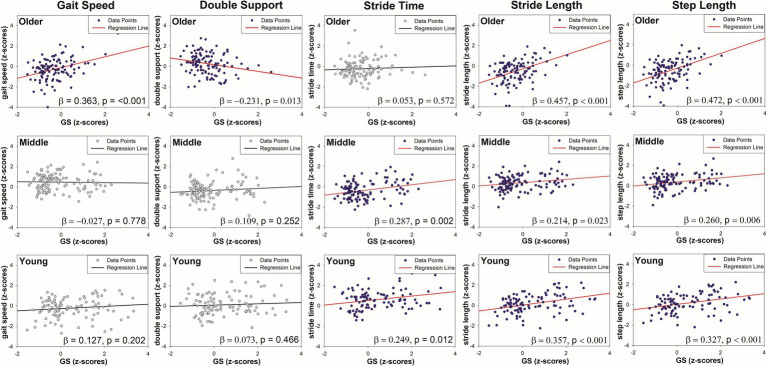
Regression plots showing age group-specific associations between grip strength (GS) and five gait parameters with significant GS by age interactions.

The body composition was determined using a bioelectrical impedance analysis (BIA) device (In body 370S, Inbody Co., Ltd., Seoul, Korea) (Figure 1A). Bioelectrical impedance analysis is a convenient method for estimating body components, which provides key basic variables such as percent body fat (%body fat), skeletal muscle mass (SMM), and body mass index (BMI), which are standard in traditional BIA devices. The BIA has been considered objective and reliable technique (27). In this study, soft lean mass, percent body fat, and BMI were retrieved from the bioelectrical signals assessed using the BIA and used as independent variables. Each variable was chosen on the basis of previous research that assessed the BIA differences in walking ability due to changes in body fat mass, muscle mass, and BMI (28).

### 2.3 Gait measurement

This study used two 7D (3D accelerometer, 3D gyroscope, and 1D barometer) inertial measurement units (Physilog 5, Gait Up, Lausanne, Switzerland), which had previously been validated as adequate for measuring mobility in stroke patients (3). The data from two 7D inertial measurement unit sensors mounted on the participants’ shoes were transferred to the analysis software and saved as spreadsheet files. To reduce experimental variability, the data from the first three phases were removed.

The 6-min walk test, a robust and repeatable assessment method that correlates with functional performance in older persons, was conducted in an indoor gymnasium at a self-selected walking speed (Figure 1B). The participants wore two Physilog5® inertial measurement unit sensors on their shoes, and only the data from the right foot were analyzed. The walking distance was set at 30 m, with cones indicating the beginning and end locations of each turn. Moreover, the participants walked back and forth for 6 min, with an average of 335.79 ± 26.91 gait cycles.

The sensor data were evaluated using the manufacturer’s software on a Lenovo T520 laptop. The measured gait variables comprised the average and CV for speed and spatiotemporal factors chosen from prior studies. The mean values and coefficients of variation represent gait performance and variability, respectively.

### 2.4 Grip strength

A hand dynamometer (Jamar, US) and hydraulic pinch gauge (Jamar, US) were used (29). The participants’ power grip and lateral pinch grip were measured three times each. The average value of the three measurements was recorded as the final data. The power grip was measured in a seated position with the elbow flexed at a 90° angle. The grip sensor was adjusted so that the second joints of the four fingers, excluding the thumb, formed a right angle (Figure 1C). The lateral pinch grip measured the force between the thumb’s pad and the lateral side of the middle phalanx of the index finger.

### 2.5 Statistics

First, the Kolmogorov–Smirnov test of normality was performed, and the physical characteristics, body composition, grip strength, and gait parameters of the young, middle-aged, and old-aged groups were compared using the one-way ANOVA and Mann–Whitney test. In the Spearman correlation analysis, after extracting the independent variable that showed a significant correlation with the dependent variables, gait performance, and gait variability, to avoid multicollinearity, the variance inflation factors were estimated for all independent variables. All data were converted to Z-values (normalized) before being entered into the regression model.

The first stage of this reciprocal analysis applied a hierarchical linear regression model for each dependent variable (i.e., average gait speed, stride length, step length, stride time, double support phase (%), and swing width) separately. Each model assesses the direct effects of the two independent variables, grip strength and age group, on a single dependent variable, while controlling for multiple extraneous variables as covariates. This repetitive approach improves the specificity and validity of the analysis for each aspect of gait performance.

**Figure 2:**
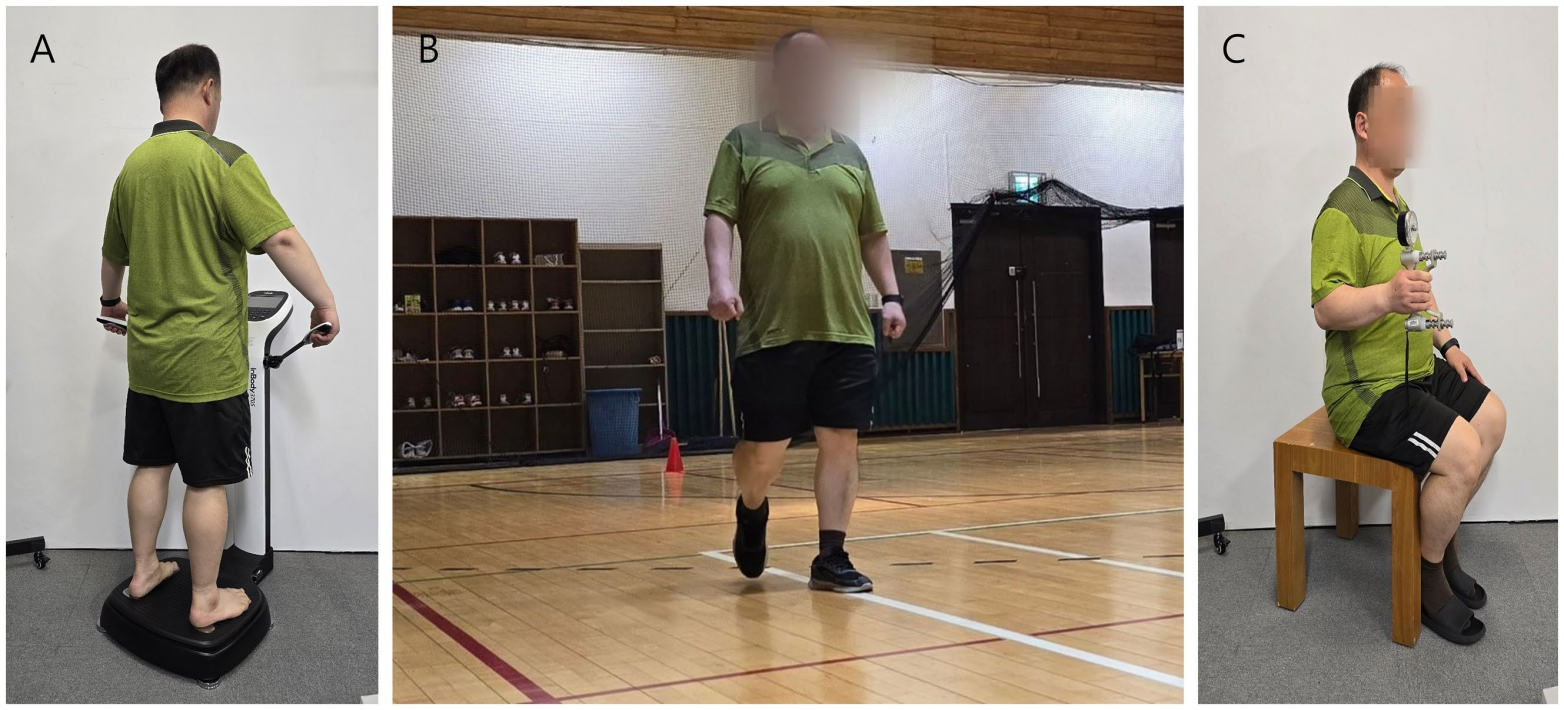
Experimental protocol: **(A)** body composition measurement, **(B)** gait assessment, and **(C)** grip strength test.

In this case, height and age were considered as the control and moderator variables, respectively. First, in Model 1 of the hierarchical moderated regression analysis, the independent variables (grip strength and age group) and the control variable (height) were input. Subsequently, in Model 2, the moderator variable (age) was included. Moreover, in Model 3, the interaction variables (BFP × age, and SMM × age, respectively) were added. Finally, if there was an interaction effect (i.e., a significant moderating effect) from Model 3, the young and older male groups were separated, and each linear regression analysis was performed and presented as a graph (Figure 2). Statistical significance was set at a *p*-value of <0.05, and SPSS 23 (IBM Inc., Armonk, NY, USA) was used for statistical analysis.

The second stage of this reciprocal analysis applied a hierarchical linear regression model separately for each alternative dependent variable: the CV values of gait speed, stride length, step length, stride time, double support phase (%), and swing width. Each model evaluates the direct effects of two independent variables, grip strength and age, on a single dependent variable, which estimates the gait variability. With reference to earlier population-based research on physical activity and health, several extraneous factors were included as covariates in each model to improve its validity (30, 31).

The third stage of this reciprocal analysis used a stepwise linear regression model to evaluate the direct effects of the average gait parameters, age, and their interaction terms on the dependent variable, which was grip strength. Multiple extraneous variables were included as covariates to improve the model’s validity and robustness.

The fourth stage of this reciprocal analysis performed a stepwise linear regression analysis to investigate the direct effects of the CV values of gait parameters and age, and their interaction terms on the dependent variable, grip strength, while controlling for multiple extraneous variables as covariates to enhance the model’s validity.

### 2.6 Covariates

According to previous cross-sectional studies (32–34), to ensure robust and unbiased results, this analysis was controlled for a range of extraneous variables as covariates. Specifically, age, sex, and physical activity levels, SMM, and body fat percentage (BFP) were included as covariates, given their potential influence on the dependent variable. Additional factors such as BMI, comorbidities, and socioeconomic status were also considered to account for the confounding effects. These covariates were carefully selected based on their established relevance in previous research and their potential to influence the relationship between the independent and dependent variables.

Statistical significance was set at a *p*-value of <0.05, and statistical analyses were performed using SPSS 27 (IBM Inc., Armonk, NY, USA). The required sample size for multiple regression analysis was determined using G*Power 3.1.9.4 (Heinrich Heine University Düsseldorf, Düsseldorf, Germany), with a significance level (α) of 0.05, statistical power (1-β) of 0.80, and a medium effect size (f^2^) of 0.15. As a result, the optimal sample size was estimated to be 107 participants.

In the regression model, the effect size was calculated as Cohen’s f^2^, with values categorized as small (0.02–0.14), medium (0.15–0.34), and large (≥ 0.35) (35). Given the sample size of this study (328 participants), the recalculated statistical power was 99%, substantially exceeding the required sample size and confirming a very high level of statistical power. This enhances the reliability of the study findings.

## 3 Results

### 3.1 ANOVA

The demographic analysis results (Table 1) revealed significant differences in sex, age, height, and weight among the young, middle-aged, and old-age groups (*p* < 0.01). With regard to physical characteristics, significant differences in handgrip strength (GS), BFP, and SMM (*p* < 0.01) were observed between the three groups, while no significant differences in their BMI were observed.

With regard to gait performance, significant differences in gait speed, double support phase, stride time, stride length, and step length were observed among the age groups (*p* < 0.01). However, no significant differences in swing width were observed. In terms of gait variability, significant differences in the double support phase CV and stride time CV (p < 0.01) were observed, while the stride length CV, step length CV, and swing width CV showed no significant differences.

### 3.2 Regression analysis of GS and age on gait performance

#### 3.2.1 Gait speed

Table 2 presents the results of the hierarchical moderated regression analysis for predicting gait speed. In Model 1, GS did not significantly influence the gait speed. In Model 2, GS still showed no significant effect, but age significantly influenced gait speed (R^2^ = 0.114, *p* < 0.01). In Model 3, the interaction between GS and age (GS × Age) was found to significantly influence gait speed (R^2^ = 0.153, p < 0.01). The detailed results for the interaction effect are illustrated in the Figure 2. Of note, of the older adults, the gait speed increased significantly with greater GS (older adults: R^2^ = 0.131, *p* < 0.001). Conversely, among the young and middle-aged adults, the changes in gait speed with increasing GS were minimal.

**Table 2:** Summary of the results of the hierarchical moderated regression analysis for predicting gait speed mean (m/s), stride time mean (s), double support phase (%), stride length mean (m), and step length mean (m).

Total (*n* = 328)					
Predictors	Gait speed β (*p*)	Stride time β (*p*)	Double support β (*p*)	Stride length β (*p*)	Step length β (*p*)
Dummy Male	−0.432 (<0.001^***^)	0.233 (0.019*)	0.326 (0.002**)	−0.364 (<0.001***)	−0.322 (<0.001***)
Height (cm)	0.385 (<0.001***)	0.296 (0.009**)	−0.176 (NS)	0.733 (<0.001***)	0.709 (<0.001***)
Weight (kg)	−0.069 (NS)	−0.036 (NS)	0.043 (NS)	−0.153 (NS)	−0.140 (NS)
BFP (%)	−0.159 (NS)	0.203 (0.034*)	0.191 (NS)	−0.054 (NS)	−0.060 (NS)
Grip Strength	0.254 (0.007**)	−0.061 (NS)	−0.143 (NS)	0.252 (0.002**)	0.237 (0.004**)
Age (years)	0.156 (0.011*)	−0.238 (<0.001***)	−0.023 (NS)	0.026 (NS)	0.026 (NS)
Age × Grip Strength	−0.138 (<0.001***)	−0.214 (0.011*)	0.197 (<0.001***)	0.169 (0.001***)	0.197 (<0.001***)
Coefficient of Determination					
Model 1 $R^{2}\left(\Delta R^{2}\right)$	0.095 (0.081)	0.167 (0.154)	0.061 (0.046)	0.343 (0.333)	0.337 (0.327)
Model 2 $R^{2}\left(\Delta R^{2}\right)$	0.114 (0.097)	0.209 (0.194)	0.061 (0.044)	0.344 (0.332)	0.338 (0.326)
Model 3 $R^{2}\left(\Delta R^{2}\right)$	0.153 (0.134)	0.225 (0.208)	0.099 (0.079)	0.367 (0.354)	0.370 (0.356)

GS, grip strength; BFP, body fat percent; $\Delta R^{2}\text{,}$ R-square change; *p < 0.05, **p < 0.01, *p < 0.001.

#### 3.2.2 Stride time

As presented in Table 2, GS did not significantly influence the stride time in Model 1. Similarly, Model 2 showed no significant effect of GS; however, age significantly influenced the stride time (R^2^ = 0.209, p < 0.001). In Model 3, the interaction between age and GS significantly influenced the stride time (R^2^ = 0.225, *p* < 0.01). The interaction effect, illustrated in the Figure 2, showed that stride time increased with greater GS among young and middle-aged adults (young adults, R^2^ = 0.062, *p* < 0.05; middle-aged adults, R^2^ = 0.082, p < 0.01). However, for older adults, increased GS did not result in significant changes in the stride time.

#### 3.2.3 Double support phase

As shown in Table 2, neither Model 1 nor Model 2 indicated a significant effect of the GS on the double support phase. However, in Model 3, the interaction between GS and age was found to significantly influence the double support phase (R^2^ = 0.099, *p* < 0.001). Among the older adults, an increase in GS was associated with a significant decrease in the double support phase (older adults: R^2^ = 0.053, *p* < 0.05). In contrast, for the young and middle-aged groups, the changes in GS did not result in significant variations in the double support phase (see Figure 2).

#### 3.2.4 Stride length

According to Table 2, GS was a significant predictor of stride length in both Model 1 (R^2^ = 0.337, *p* < 0.001) and Model 2 (R^2^ = 0.338, *p* < 0.001). In Model 3, the interaction between GS and age significantly affected the stride length (R^2^ = 0.370, *p* < 0.001). Across all age groups, an increase in GS was associated with a significant increase in stride length (young adults, R^2^ = 0.127, *p* < 0.001; middle-aged adults, R^2^ = 0.046, *p* < 0.05; older adults, R^2^ = 0.209, *p* < 0.001) (see Figure 2).

#### 3.2.5 Step length

Table 2 shows the regression analysis results predicting the step length. Similar to the stride length, GS was a significant predictor of step length in both Model 1 (R^2^ = 0.337, p < 0.001) and Model 2 (R^2^ = 0.338, p < 0.001). In Model 3, the interaction between GS and age significantly affected the step length (R^2^ = 0.370, p < 0.001). An increase in GS led to a significant increase in step length across all age groups (young adults, R^2^ = 0.107, p < 0.001; middle-aged adults, R^2^ = 0.068, *p* < 0.01; older adults, R^2^ = 0.222, p < 0.001) (see Figure 2).

### 3.3 Regression analysis of GS and age on gait variability

The GS did not significantly affect the swing width, stride time CV, step length CV, or double support phase CV. Age was identified as the only significant predictor of these variables. Furthermore, the regression models for swing width CV and stride length CV were not statistically significant.

### 3.4 Regression analysis of mean gait variables and age on grip strength

The regression analysis of gait variability variables and age on grip strength revealed no significant effects from the gait variability variables or interaction terms. In the final model, only SMM, gender, and age remained significant predictors (R^2^ = 0.688, *p* < 0.001).

### 3.5 Regression analysis of gait variability and age on grip strength

**Figure 3:**
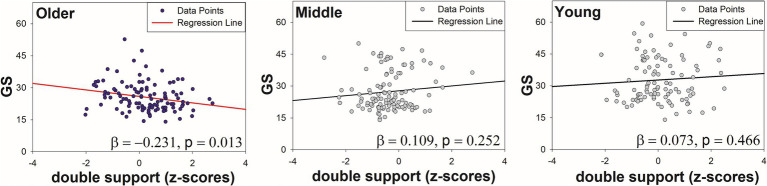
Regression plots showing the association between double support phase and GS by age group.

Table 3 shows the regression analysis results of the mean gait variables and age on grip strength. In the final model (Model 4), the interaction term between age and double support phase mean significantly influenced the grip strength (R^2^ = 0.696, *p* = 0.016). Of note, in the older adult group, an increase in the double support phase mean was associated with a tendency for a weaker grip strength (older adults: R^2^ = 0.231, *p* < 0.05) (Figure 3). In contrast, no significant associations were observed in the young group (R^2^ = 0.005, *p* = 0.466) or middle-aged group (R^2^ = 0.012, *p* = 0.252).

**Table 3:** Summary of the results of the stepwise regression analysis for predicting grip strength (kg).

Predictors	Total (*n* = 328)							
	Grip strength							
	Model 1		Model 2		Model 3		Model 4	
	β	*p*	β	*p*	β	*p*	β	*p*
SMM	0.810	<0.001***	0.566	<0.001***	0.516	<0.001***	0.505	<0.001***
Dummy_male			0.292	<0.001***	0.309	<0.001***	0.314	<0.001***
Stride Length mean					0.099	0.003**	0.094	<0.001***
Age × DS mean							−0.075	0.016*
$R^{2}$ block 1 = 0.810 $\Delta R^{2}$ = 0.657; $R^{2}$ block 2 = 0.826 $\Delta R^{2}$ = 0.682; $R^{2}$ block 3 = 0.831 $\Delta R^{2}$ = 0.691; $R^{2}$ block 4 = 0.834 $\Delta R^{2}$ = 0.696.								

SMM, Skeleton Muscle Mass; DS, Double Support; ∆R², R-square change; *p < 0.05; **p < 0.01; *p < 0.001.

## 4 Discussion

This study explored the reciprocal relationship between grip strength and gait function, emphasizing the bidirectional effect of age on gait performance and variability. Compared with previous studies that have primarily examined the effect of grip strength on walking ability, this study also investigated how gait function influences grip strength. To identify age-specific intervention targets, the interactive influence of grip strength and age on spatiotemporal gait parameters and variability was assessed. The findings confirm a significant interaction between age and grip strength, revealing that age moderates the relationship between muscular strength and gait function. Furthermore, this study uniquely incorporates gait variability as a major component to address the literature gap. We hypothesized that older adults with reduced grip strength would exhibit poorer gait performance and greater gait variability than younger and middle-aged adults.

### 4.1 Grip strength and gait performance

Lower grip strength was associated with poorer gait performance, and this trend was more significant in older adults. Specifically, spatiotemporal gait variables such as gait speed and spatial variables like stride length and step length decreased as grip strength weakened, regardless of age, but the effects were significantly greater in the older adults. The regression analysis revealed that the explanatory power of grip strength for gait speed was 1.6% in young adults, 0.1% in middle-aged adults, and 13.1% in older adults. For stride length, the values were 12.7, 4.6, and 20.9%, respectively, and for step length, 10.7, 6.8, and 22.2%, respectively.

The temporal variables such as stride time and double support phase tended to increase as grip strength decreased, regardless of age, but the effects were significantly greater in the older adults. The regression analysis revealed that the explanatory power of grip strength for stride time was 6.2% in young adults, 8.2% in middle-aged adults, and 0.3% in old-aged adults, while for double support, the values were 0.5, 1.2, and 5.3%, respectively.

The relationship between grip strength and gait performance has been consistently reported in previous studies. For example, Stevens et al. (36) found that a stronger grip strength was associated with a faster gait speed and better physical performance. Lin et al. (37) and Felix et al. (38) also identified grip strength as a useful surrogate marker for identifying slow gait speed in older adults. These studies suggest that grip strength reflects the overall functional capacity and plays a critical role in predicting frailty and mobility decline. Jabbar et al. (39) reported that with age, both grip strength and lower limb strength decline, and grip strength significantly affects the major spatiotemporal gait parameters such as gait speed, stride length, stride time, and double support time.

The findings of this study aligned with those of previous studies, validating that aging is associated with a decline in grip strength and spatiotemporal gait parameters. According to Hortobágyi et al. (40), older adults tend to compensate for reduced ankle strength by relying more on the hip muscles during walking. As grip strength tends to decrease with age, age-related differences in the relationship between grip strength and gait may be related to such muscular changes. Previous studies have also indicated the association of grip strength with broader functional indicators, such as muscle mass index and sleep health, further supporting its role as a biomarker for overall functional capacity (41, 42). Moreover, grip strength may reflect neuromuscular control efficiency, which is critical for coordinated movements, including gait performance (43). Age-related decline in neuromuscular control has been linked to low motor unit recruitment and firing rates (44, 45), which could affect handgrip and lower limb muscle function, thereby influencing gait performance (43, 46). These findings emphasize the importance of grip strength not only as an indicator of upper body strength but also as a biomarker of overall muscle health and functional capacity (47). These results confirm that the effects of grip strength on gait performance vary with age, supporting previous findings indicating that changes in muscle strength due to aging play a critical role in gait performance. Age-related muscle strength decline is a major factor contributing to reduced physical function and independence in older adults, which can lead to diminished gait performance and balance ability (48). Sarcopenia, which is prevalent among older adults, is closely associated with decreased gait speed and increased fall risk (49).

In contrast, in young and middle-aged adults, grip strength has limited explanatory power for gait performance. This may be because gait performance and physical function in these groups are maintained by a complex interplay of factors beyond muscle strength, such as balance, flexibility, and neuromuscular coordination (50, 51).

### 4.2 Grip strength and gait variability

Grip strength did not significantly influence gait variability, with age being the only significant predictor. Specifically, the stride time CV and double support CV exhibited significant differences by age, while the regression models for the spatial variability parameters were not significant.

These findings suggest that grip strength is not strongly associated with gait variability and that age itself is a more critical predictor. Previous studies, such as those by Lee et al. (52) and Ciprandi et al. (53), have suggested the potential of grip strength as a key indicator of gait variability. Lee et al. (52) also reported significant negative correlations between grip strength and variables such as stride length CV, max heel clearance CV, and max toe clearance CV. Ciprandi et al. (53) have shown that grip strength significantly influenced gait variability indices such as stride length, stride time, step width, and double support CV, indicated that a decline in grip strength was linked to increased gait variability.

Previous studies have suggested a strong association between grip strength and gait variability (52). However, this study did not find significant association when accounting for the effects of age. This finding highlights the greater effect of age on gait variability than grip strength and partially aligns with the results of this study, which examined the interaction between age and grip strength. Unlike previous studies that have analyzed limited age ranges or focused on interaction effects such as age and sex, this study investigated the interplay between grip strength and age. Furthermore, controlling for body composition variables such as SMM and BFP likely contributed to the differences in findings.

### 4.3 Gait ability, age, and their interaction with grip strength

Gait ability and age were significant predictors of grip strength, with the interaction between gait ability and age prominently reflected in the double support phase. This study suggested that the double support phase, a key gait characteristic in older adults, significantly interacted with age to predict grip strength in the regression analysis. Specifically, the grip strength decreased, while the double support time increased with advancing age.

This finding reflects the physiological and behavioral adaptations required to maintain stability and balance with aging. The double support phase, wherein both feet are in contact with the ground, plays a critical role in stabilizing the gait. Older adults often prolong this phase to ensure stability, especially in the context of declining strength and balance. Previous studies have also reported that the proportion of the double support phase increases with age and is associated with fall risk and balance deficits (54–56).

## 5 Limitations

However, this study has several limitations. First, the participants were limited to healthy community-dwelling individuals, which may restrict the generalizability of the findings to other populations. Future studies should cover other populations, such as individuals with chronic diseases or mobility impairments.

Second, although sex was included as a control variable in the regression model, the difference in the sex ratio between males and females may have had a latent effect on the overall results.

Third, nonsignificant results for certain gait parameters in the regression models may reflect the limitations in the model’s fit. Thus, future studies should incorporate additional variables, such as gait speed and other physical factors (e.g., muscle strength), to enhance the analytical framework. Moreover, as a cross-sectional study, this research cannot establish causal relationships between grip strength and gait performance or variability. Longitudinal studies are needed to investigate these relationships over time.

## 6 Conclusion

This study analyzed the influence of grip strength on gait across different age groups, indicating that grip strength is significantly associated with gait performance but not with gait variability. These findings suggest that while grip strength partially explains the functional aspects of gait, it cannot fully account for gait stability or fall risk. Therefore, it is essential to use a range of evaluation tools alongside grip strength in functional assessments and to determine whether grip strength is an appropriate standalone assessment tool for gait.

Moreover, the finding that grip strength has a greater impact on gait performance in older adults indicates that grip strength may serve as an effective measure for evaluating physical function in this population. This emphasizes the potential for using grip strength as a tool for assessing gait ability in older adults as well as the need for follow-up studies to support this application.

Finally, the key conclusions derived from this study are as follows:

1. Among older adults, greater grip strength tends to improve gait performance.
2. The influence of grip strength on gait performance differed across age groups.
3. Although grip strength does not significantly impact gait variability, gait variability tends to increase with age.
4. The proportion of the double support phase varies with age and functions as a predictive factor for grip strength.

This study provides a valuable foundation for understanding the relationship between aging and grip strength and their combined effects on gait performance and stability. The findings can contribute to the design of tailored rehabilitation and exercise intervention programs for older adults.

## Data Availability

The original contributions presented in the study are included in the article/supplementary material, further inquiries can be directed to the corresponding author.
